# Epithelial–Mesenchymal Transitions during Neural Crest and Somite Development

**DOI:** 10.3390/jcm5010001

**Published:** 2015-12-23

**Authors:** Chaya Kalcheim

**Affiliations:** Edmond and Lili Safra Center for Brain Sciences (ELSC), Department of Medical Neurobiology, Institute for Medical Research Israel-Canada (IMRIC), Hebrew University of Jerusalem-Hadassah Medical School, P.O. Box 12272, Jerusalem 9112102, Israel; kalcheim@cc.huji.ac.il; Tel.: +972-2-6758438; Fax: +972-2-6757451

**Keywords:** BMP, cell fate, cell migration, dermis, dermomyotome, FGF, N-cadherin, neural tube, sclerotome, Wnt

## Abstract

Epithelial-to-mesenchymal transition (EMT) is a central process during embryonic development that affects selected progenitor cells of all three germ layers. In addition to driving the onset of cellular migrations and subsequent tissue morphogenesis, the dynamic conversions of epithelium into mesenchyme and *vice-versa* are intimately associated with the segregation of homogeneous precursors into distinct fates. The neural crest and somites, progenitors of the peripheral nervous system and of skeletal tissues, respectively, beautifully illustrate the significance of EMT to the above processes. Ongoing studies progressively elucidate the gene networks underlying EMT in each system, highlighting the similarities and differences between them. Knowledge of the mechanistic logic of this normal ontogenetic process should provide important insights to the understanding of pathological conditions such as cancer metastasis, which shares some common molecular themes.

## 1. Introduction

In vertebrates, epithelium and mesenchyme are basic tissue phenotypes. Epithelial–mesenchymal transitions (EMT) and the reverse process, mesenchymal–epithelial transitions (MET) are critical for development of many tissues and organs in the embryo. Epithelial and mesenchymal cells differ in phenotype as well as function. Epithelial cells are closely connected to each other by tight junctions, gap junctions and adherens junctions, have an apico-basal polarity characterized by polarization of their actin cytoskeleton and a basal lamina at their basal surface. Mesenchymal cells, on the other hand, lack this polarization, have a spindle-shaped morphology and interact with each other only through focal points [1]. Neural crest (NC) cells as well as somite cells and their intermediate derivatives are excellent examples of transient embryonic subsets that undergo the above transitions.

At the population level, both NC and somites are multipotent and yield a rich assortment of derivatives. The vertebrate NC is the source of neurons, glia and Schwann cells of the peripheral nervous system (sensory, sympathetic, parasympathetic and enteric ganglia). It also generates pigment cells of the skin, chromaffin cells in the adrenal gland, and additional endocrine derivatives [2,3]. The NC is also widely believed to be the source of mesectodermal derivatives (skeletogenic, odontogenic, connective tissue and smooth muscle) in the vertebrate head [2,4,5]. However, recent data have challenged this notion and suggested instead that a lateral non-neural domain of the neural fold epithelium, termed “metablast”, is a source of ectomesenchyme distinct from the NC ([6] and refs therein).

Another salient organizational feature of vertebrate embryos is the segmental organization of the paraxial mesoderm closely aligned on both sides of the axial organs, the NT and notochord, and medially localized with respect to the intermediate and lateral plate mesoderms. Laid down during gastrulation, the unsegmented mesoderm progressively epithelializes via a process of MET and subdivides to generate somites. These are transient sphere-like structures that set the basis for the segmental organization of skeletal derivatives, as well as that of peripheral ganglia and nerves [7,8,9,10,11]. The somites develop stepwise in a rostral to caudal gradient along the body axis so that the caudalmost somites are the youngest. Subsequently, they undergo a regional process of EMT by which they dissociate ventrally to give rise to the mesenchymal sclerotome while the remaining dorsal epithelial roof becomes the dermomyotome (DM). The sclerotome generates the vertebral column, ribs, tendons and meninges [12] and the DM is the source of vertebral and limb muscles, dermis, endothelial cells, smooth muscle cells and cartilage of the scapula blade [13,14,15].

Several characteristics are shared by both systems. For instance, the dorsal region of the NT that generates NC cells and the paraxial mesoderm-derived somites are composed of pseudostratified epithelia in which progenitor cells undergo interkinetic nuclear migration and exhibit typical patterns of cell proliferation [16,17,18]. Second, these epithelia are transient as progenitor cells either delaminate progressively or fully dissociate to generate mesenchymal cells. In the dorsal NT of avians at trunk levels, EMT and cell delamination are gradual events lasting for almost two consecutive days during which the dorsal NT preserves its general epithelial structure [19]. In contrast, cranial NC cells exit the neural folds or the closed NT as a cohesive group of progenitors which undergo only a partial EMT and rapidly split into distinct streams of cells [20]. Furthermore, the regulatory networks controlling EMT at each level clearly differ (reviewed in [21,22]).

Comparable processes take place in the DM, which is composed of four contiguous lips and a central sheet. Whereas the lips produce myocytes over a few days while remaining epithelial, the central sheet completely dissociates to produce dermis and mitotic progenitors [23,24,25,26].

An additional common feature is the existence of long and short-range cellular migrations that follow initial EMT. For instance, studies performed in avian embryos revealed that NC progenitors that form sympathetic ganglia migrate a long distance from the dorsal NT toward the dorsal aorta, and then along the aorta for a length of three consecutive somites rostral and caudal vis-à-vis their exit point in the NT [27]. A similar behavior was documented for melanocytes, which migrate through the dorsal dermis before invading the ectoderm [27]. Hence, a given segment of the NT contributes NC cells to at least six consecutive sympathetic ganglia and also pigment cells to an equivalent length of skin. Sensory progenitors of the dorsal root ganglia (DRG) also move longitudinally along the basement membrane of the tube for the length of about one and a half segments before migrating dorsoventrally a short distance until reaching the DRG primordium; in this way progenitors arising at a given segmental level populate two consecutive DRG in a stereotypic pattern [28].

Likewise, the ventrolateral lip of the DM at brachial and lumbosacral levels of a variety of species undergoes EMT and Pax3/7-positive progenitors migrate a long way into the limb primordia to generate the appendicular muscles [29]. In addition, endothelial cells derived from the lateral DM of a given somite colonize blood vessels along at least two segments [15]. Different from the precedent cases, progenitors of myotomal muscles either translocate directly from the DM into the adjacent myotome and then differentiate into mononucleated, unit-length myofibers [30,31,32,33] or, as in the case of avian pioneer myoblasts, undergo a Slit1/Robo2-dependent caudal to rostral migration within a single segment followed by differentiation in the opposite direction [34]. Similarly, in zebrafish, slow twitch muscle cells derived from adaxial progenitors undertake a N- and M-cadherin-dependent migration from the medial notochord to the lateral aspect of the myotome within the same segment [35].

Altogether, NC and dorsal somite progenitors share many similar traits during both epithelial and mesenchymal states. Nevertheless, heterogeneity in EMT patterns and migratory behavior are apparent within each system along the axis and at various stages. Undoubtedly, in both systems, EMT is a prerequisite and a driver for further cell migrations, events that are essential for proper morphogenesis. Since the above ectodermal and mesodermal progenitors yield a rich variety of derivatives, a fundamental question is whether the epithelia of origin are composed of naïve precursors or, alternatively, of fate-restricted cells. In the former case, one would postulate that cell fate decisions occur during migration or at the homing sites thus imposing the search for responsible mechanisms that operate after EMT. In the latter event, research should focus on mechanisms operating within the epithelium itself that would account for the emergence of an early heterogeneity. Hence, the epithelial–mesenchymal status of these early precursors may directly or indirectly be associated with the acquisition of final cell identities, as discussed elsewhere [36].

## 2. EMT of Neural Crest Cells

### 2.1. NT–Somite Interactions Underlie NC EMT in the Trunk

**Figure 1:**
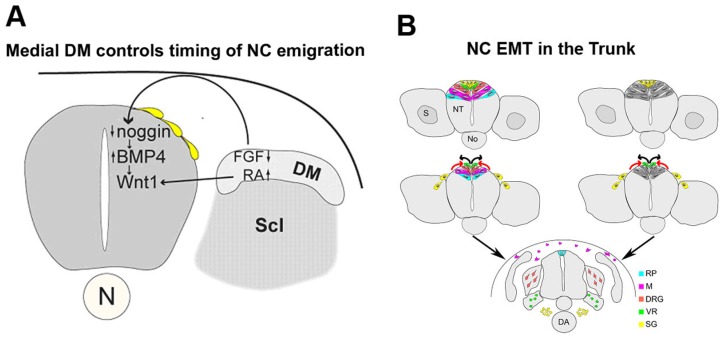
Epithelial to mesenchymal transition (EMT) of neural crest (NC) progenitors: Regulation and cellular dynamics. (**A**) The medial lip of the nascent dermomyotome (DM) controls the timing of NC delamination. In the early dorsal NT, prior to the onset of NC emigration, levels of Noggin are high thereby inhibiting the activity of BMP4 and NC delamination. The forming medial lip of the DM acts upon the dorsal NT via FGF8 and retinoic acid signaling to inhibit local noggin transcription thus relieving BMP4 which stimulates NC emigration. Scl, sclerotome; (**B**) Two possible models representing the dynamic dorsalward relocation of NC cells prior to emigration in association with fate restriction. In both models, the emigrating cells are largely fate-restricted. In the model on the left (spatial mechanism), a pattern reflecting the different fates is already apparent in the dorsal NT (color coding). In the model depicted on the right, fate restriction is assigned to a cell upon relocation to the dorsal midline area by a time-dependent mechanism. Abbreviations: DRG, dorsal root ganglia; M, melanocytes; RP, roof plate; SG, sympathetic ganglia; VR, Schwann cells along ventral root.

A significant body of evidence, primarily stemming from avian embryos, relates the onset of NC migration in the trunk with somitogenesis and subsequent somite dissociation [28,37]. At the level of the segmental plate, presumptive NC progenitors are still confined to the dorsal NT [28]. Emigration of the first NC cells becomes apparent at levels opposite epithelial somites. Furthermore, upon somite dissociation, NC cells continue exiting the neuroepithelium and begin simultaneously invading the somite in a segmental fashion (Figure 1B) [28,37,38,39]. This suggested that the paraxial mesoderm regulates aspects of NC EMT and emigration. However, in the rostral trunk, signals triggering NC EMT may not be under the strict control of the somites as at early stages, there is an asynchrony between somitogenesis and neural crest departure [40]. As briefly introduced above, cranial NC delamination and EMT obey distinct rules as the cranial mesoderm is not segmented. Cellular and molecular (transcriptional and epigenetic) aspects of cranial NC behavior are being elucidated and the reader is referred to interesting articles and reviews covering this topic [22,41,42,43].

This review focuses on trunk levels of the neuraxis and only briefly discusses the process of EMT at additional levels for comparison. In the trunk region, an interplay between noggin and BMP4 in the dorsal NT was found to generate a graded activity of the latter that, via regulation of *Wnt1* transcription and Wnt-dependent canonical signaling, triggers EMT of NC progenitors and the consequent onset of cell migration [44,45]. This rostral-to-caudal gradient of BMP4 activity is generated in spite of a constant level of BMP4 mRNA production along the dorsal NT by virtue of an opposing, decreasing gradient of *noggin* transcription and activity that correlates with somite development. Downregulation of *noggin* progressively relieves inhibition of BMP and allows NC EMT (Figure 1A). The mechanism by which this graded expression of *noggin* is generated in the dorsal NT remained unclear. Due to the temporal correlation between *noggin* levels and somite development, somitic factors were suggested to influence the levels of *noggin* mRNA in the NT. Consistent with this notion, grafting experiments revealed that dissociating somites emit an inhibitor of *noggin* production in the dorsal NT thereby coupling the time of EMT with the development of the somites as suitable substrates for subsequent NC migration [46]. The identity of this factor(s) remained unknown for years. More recently, opposing gradients of FGF and retinoic acid, apparent in the paraxial mesoderm, were reported to control the timing of NC EMT, in addition to affecting specific aspects of NC induction. A decrease in FGF at somitic levels was suggested to be required for *noggin* downregulation. In contrast, retinoic acid was not necessary for modulating *noggin* transcription. In addition, FGF signaling prevented the premature expression of *Wnt1*, whereas retinoic acid triggered *Wnt1* transcription at axial levels containing specified NC progenitors (Figure 1A). Hence, counter-gradients of FGF and retinoic acid affect NC EMT in part through the modulation of specific aspects of the BMP and Wnt signaling pathways [47], previously shown to set in motion the process (see above).

### 2.2. Mechanisms of Trunk NC EMT that Operate Downstream of BMP Signaling

Further insights into this process stemmed from the discovery that a large proportion of trunk NC cells synchronize to the S-phase of the cell cycle during EMT, and that the transition from G1 to S is necessary for NC EMT [17]. Furthermore, these mechanisms are hierarchically related as BMP acts upstream of canonical Wnt1 activity, which in turn promotes G1/S transition and NC delamination [44]. Notably, cranial NC cells do not synchronize to S the phase by the time of EMT, another feature distinguishing cranial from trunk NC behavior which is likely related to the observation that EMT during delamination from the cranial neuroepithelium is only partial and that different genes operate at each axial level [48].

Aiming to understand the function of effectors operating in the trunk downstream to BMP/Wnt, it was shown that in the dorsal NT at premigratory levels, membrane-associated N-cadherin is strongly expressed, contributing to preservation of the epithelial state of presumptive NC cells both by a cell adhesion-mediated mechanism as well as by inhibiting β-catenin-dependent Wnt signaling [49]. When noggin activity is downregulated and BMP is consequently activated, N-cadherin is proteolytically degraded via a BMP and ADAM10-dependent mechanism with no apparent change at the transcriptional level. This leads to the formation of a soluble fragment, CTF2, the end product of N-cadherin degradation. CTF2 translocates into the nucleus, stimulates transcription of cyclinD1, an important regulator of G1/S phase transition, which together with CDK4/6, promotes cell cycle progression, altogether inducing NC EMT [49]. In general agreement with the precedent results, live cell confocal time-lapse imaging of explanted neural primordia revealed that in most EMTs, the apical cell tail is indeed cleanly retracted from the lumen of the neuroepithelium with a concomitant downregulation of N-cadherin, followed by movement of the cell body out of the NT. However, in exceptional cases, the rupture of the NC cell tail during retraction was observed before junctional complexes were completely downregulated [50], indicating possible mechanistic variability, or, alternatively, reflecting differences between the behavior of NC cells *in ovo* compared to tissue explants.

An additional cadherin expressed in avian premigratory NC is Cad6B. Previous studies documented a functional role for Cad6B in NC EMT at both cranial [51] and trunk [52,53] levels. Overexpression of Cad6B in the dorsal NT at a cranial level leads to a disruption in NC migration, with a concomitant aggregation of cells adjacent to the NT [51,52]. Reciprocally, Cad6B knockdown results in premature exit of NC cells, suggesting a role for Cad6B levels in cranial NC cells undergoing *en masse* EMT [51]. Like N-cadherin, Cad6B is rapidly depleted from premigratory NC cells [49,54]. This is accounted for by its proteolytic processing occurring at the level of the midbrain [54], similar to what has been previously demonstrated for N-cadherin in the trunk [49]. Taken together, these findings underscore the importance of post-transcriptional processing of adhesion molecules in the generation of migratory NC cells along the axis. To note is that, in contrast to the results discussed above, other studies showed that signaling through Cad6B in fact positively mediates NC de-epithelialization, loss of cell polarity and increased migratory ability in trunk NC cells suggesting that Cad6B promotes EMT [53,55]. In agreement with these results, Clay and Halloran [56] used live imaging of NC behavior in zebrafish embryos and documented that Cad6 promotes detachment of apical NC tails, an important early step of EMT. In this context, Cad6 affected the spatiotemporal dynamics of F-actin and was required for its accumulation in apical NC tails during detachment while affecting the distribution active Rho, which is known to promote localized actomyosin contraction in detaching cells [56].

Rho GTPases are important and well documented contributors of EMT and motility as they control the dynamics of the actin cytoskeleton, cell polarity, gene transcription, cell cycle progression, *etc.* [57]. In different contexts, Rho signaling has been shown to promote cell migration or, conversely, to maintain the epithelial state [58,59,60,61,62,63]. In the trunk NC of avian embryos, Rho through Rho-associated kinase (Rock) activity, negatively modulates NC EMT while acting downstream of BMP and of G1/S transition, maintaining the integrity of N-cadherin-mediated apical junctions and a stable cytoskeleton composed of actin stress fibers [64]. In the zebrafish hindbrain, imaging of endogenous active Rho revealed that Rho is activated in a discrete apical region of premigratory NC cells during EMT, and that Rho-ROCK signaling is essential for apical detachment and for generation of motility within the neuroepithelium [63]. Taken together, the precedent results describe possible and alternative mechanisms by which specific cadherins or Rho-GTPases can generate cell motility during EMT. Given the dynamic nature of the EMT process, it is important to consider contextual differences such as species, axial level specificity, and the subcellular resolution under assessment, in evaluating the precise function of these important cell motility modulators [65].

Activation of another Rho-GTPase, Rac, leads to the assembly of a dynamic meshwork of actin filaments at the cell periphery that produce lamellipodia and membrane ruffles [66]. In migrating cells, Rac is required at the front of the cell to regulate actin polymerization and membrane protrusions that enable cell motility. For efficient cell migration, this activity is spatially restricted with highest concentrations at the leading edge [67,68]. On the other hand, in migrating cells, Rho is thought to regulate the contraction and retraction forces required in the cell body and at the rear, and would thus be expected to follow an inverse distribution to that of Rac. In various systems, ranging from fibroblasts to neurons, the activities of Rho and Rac seem to be antagonistic (see for example [69]). This also applies for the NC, where the promotor activity of *Xenopus* Snail2 is enhanced by either inhibition of Rho or activation of Rac and *vice*-*versa* [70]. Rho and Rac were also found to exert reciprocal effects on directional migration of cranial NC cells in *Xenopus* that is mediated by syndecan-4 and by the planar cell polarity pathway [71,72,73]. At the early stages of trunk NC ontogeny, Rac was found to be necessary for NC migration but dispensable for earlier cellular EMT [74]. In addition, Rho activity that maintains NC cells in the epithelial state, is inhibited during EMT upon loss of N-cadherin-mediated adhesions [64] and this inhibition of Rho stimulates Rac1 activity and consequent formation of lamellipodia that enable further cell migration. Hence, in the context of avian NC EMT, delamination and migration, the activities of Rho and Rac are differential, sequential and antagonistic.

### 2.3. Sequential EMT of NC Cells Is Associated with the Colonization of Peripheral Targets and with Fate Restrictions

Spatial and temporally controlled lineage analysis performed in avian embryos at very precise time points shows that NC cells that exit the neural primordium following an EMT first migrate towards the dorsal aorta where they become sympathetic ganglia. The next in line to emigrate generate Schwann cells, then cells in dorsal root sensory ganglia and finally melanocytes of the skin. Following the end of NC emigration, the dorsal midline of the NT becomes the roof plate, a signaling center for the organization of dorsal neuronal cell types [19,75]. Most importantly, this ordered delamination of NC cells is accompanied by a ventral to dorsal relocation of the premigratory progenitors prior to leaving the NT [19,76]. In this regard, prospective roof plate cells were shown to originate ventral to presumptive NC and to progressively relocate dorsalward to occupy their definitive midline position following crest delamination (Figure 1B). This lineage analysis of premigratory progenitors, coupled to challenging their migratory environment, also revealed that the dorsal NT is a highly dynamic region sequentially traversed by fate-restricted crest progenitors [19,77]. These data raise important questions regarding the mechanisms of cell emigration in relation to fate acquisition, and suggest the possibility that spatial and/or temporal information in the dorsal neural tube determines initial segregation of NC cells into their derivatives (Figure 1B).

## 3. Cell Rearrangements during Somite Development

Sequential and reiterative events of MET and EMT are an amazing feature of paraxial mesoderm development that exquisitely illustrate the significance of these cellular rearrangements to proper formation of vertebrae, dermis and muscles. These are discussed in the next few sections.

### 3.1. Somite Epithelialization—A Mesenchymal-to-Epithelial Transition (MET)

The paraxial mesoderm arises during gastrulation as columns of mesenchymal progenitors, the segmental plate, laid down on both sides of the midline. Next, the segmental plate undergoes two critical events that progress in a rostral to caudal direction along the axis and that culminate with the gradual formation of metamerically arranged somites; the formation of intersomitic boundaries and a process of MET. The temporal association of intersomitic border formation and somite epithelialization suggests a common regulation, yet the two processes are separable. This is clearly exemplified in the Paraxis mutant mice. Paraxis is a bHLH transcription factor required for somite epithelialization. In Paraxis mutants, the intersomitic borders form normally, but no epithelialization takes place, resulting in mesenchymal blocks instead of epithelial somites. Surprisingly, the mutant embryos develop somite derivatives such as muscle and sclerotome, although the vertebrae and dorsal root ganglia are fused [78]. Notably, different parts of the somite epithelialize at different time points; the future medial and lateral walls of the epithelial somite begin to epithelialize already in the anterior PSM [79]. In chicken, the posterior somitic wall begins to epithelialize before the anterior wall [80], altogether suggesting a complex regulation by signals emanating from tissues adjacent to the somite boundaries.

Consistent with this notion, somite epithelialization was shown to depend on exogenous factors. Removal of the ectoderm over the segmental plate abolishes epithelialization and *paraxis* expression in all but the dorso-medial corner of the somite, adjacent to the NT. Insertion of an impermeable barrier between the caudal segmental plate and the NT/notochord also prevents epithelialization and abolishes *paraxis* expression. Wnt6, normally expressed in the ectoderm, rescues the effects of both ectoderm removal and detachment from the NT, while restoring *paraxis* transcription. Wnt6 overexpression prolongs the epithelial state of the somite and delays differentiation. Implantation of beads soaked with Sfrp-2, a soluble Wnt antagonist, inhibits somite epithelialization, with corresponding effects on *paraxis*. These findings indicate that ectodermal Wnt6 and perhaps additional NT-derived Wnts are required for the epithelialization of somites from the segmental plate, and function upstream of Paraxis [81]. Recently, a genome-wide comparison of gene expression in the anterior segmental plate and newly formed somites of Paraxis mutant embryos showed an enrichment of factors associated with extracellular matrix assembly, cytoskeletal organization and intercellular and cell-matrix adhesion. The greatest change in expression was seen in fibroblast activation protein alpha encoding a dipeptidyl peptidase capable of increasing fibronectin and collagen fiber organization in extracellular matrix. Further, downstream genes of the Wnt and Notch signaling pathways were downregulated, confirming that Paraxis and Wnt pathways cooperate and further suggesting interactions between Paraxis and Notch components [82].

In contrast to avians and mice, development of the paraxial mesoderm in *Xenopus laevis* is different because it does not form an epithelial somite. Both gain and loss of function of Paraxis were shown to affect somite elongation, rotation and alignment, causing a severe disorganization of the somitic tissue. Furthermore, manipulation of Paraxis levels affected expression of cell adhesion markers as well as proper expression of myotomal and sclerotomal differentiation traits [83].

As found to be the case for the NC, Rho GTPases are effectors of EMT and MET also in somites. Plasmids coding for constitutively active or dominant negative forms of Cdc42 and Rac-1 were electroporated into the chick segmental plate mesoderm. Cells expressing either constitutively active Cdc42, dominant negative Rac-1 or constitutively active Rac-1, failed to epithelialize. In contrast, inhibition of Cdc42 activity led to a hyper-epithelial phenotype. Thus, Cdc42 activity is necessary for development of mesenchymal properties. In addition, proper levels of Rac1 are required for somite epithelialization. The latter is also reflected in the observation that cells overexpressing active Rac-1 had upregulated but unpolarized levels of N-cadherin. Also, Rac-1 activity was found to be required for the epithelializing activity of Paraxis [80]. Hence, the details of the gene regulatory cascade underlying somite epithelialization begin to emerge, and involve ectodermal-derived Wnts acting upstream of Paraxis which may also feed-back on to Wnt signaling, and specific Rho-family GTPases. Downstream effectors are likely to be cadherins and selected extracellular matrix molecules directly responsible for the establishment of epithelial features.

### 3.2. Somitic EMT: The Sclerotome and Its Subdomains

**Figure 2:**
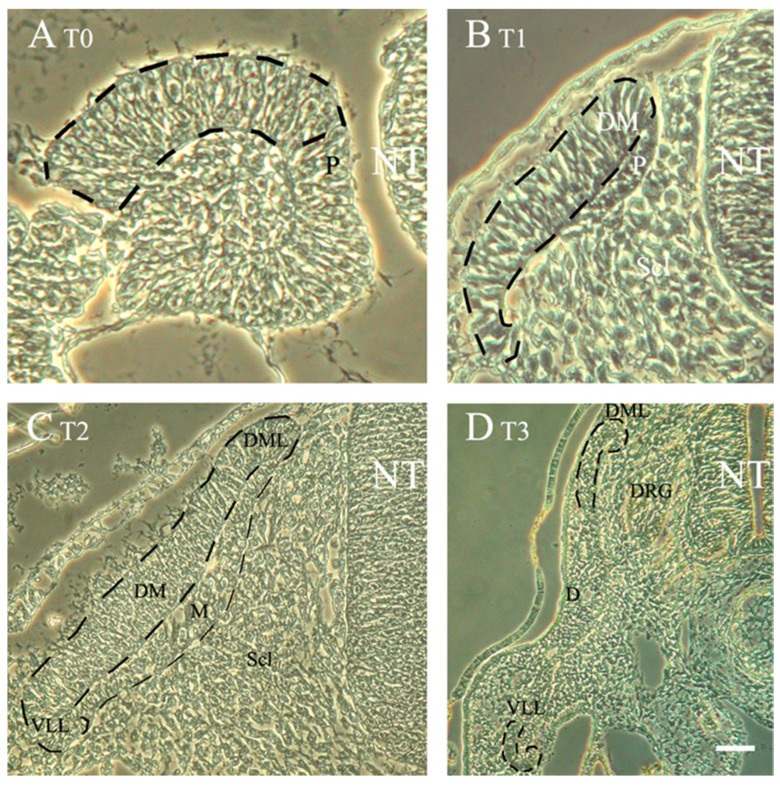
Successive stages in dermomyotome development at flank levels of the axis. Phase contrast images that illustrate: (**A**) T0, epithelial somite stage with the prospective dermomyotome (DM) highlighted by dashed lines; (**B**) T1, initial formation of the DM following mesenchymalization of the sclerotome (Scl). At this stage the pioneer (P) myoblasts bend underneath the nascent DM; (**C**) T2, the DM of a fully dissociated somite in which the primary myotome (M) is well differentiated. Note the well defined medial and lateral edges (DML and VLL, respectively); (**D**) T3, the DM dissociates into dermis except for the DML and VLL which still remain epithelial (demarcated by dashed lines). See text for precise stages. Abbreviations, DRG, dorsal root ganglion, NT, neural tube. Bar = (**A**) 8 μM; (**B**) 15 μM; (**C**) 22 μM; and (**D**) 80 μM.

Ventromedial cells of the epithelial somite express *Pax1* and generate the sclerotome by undergoing a remarkable process of EMT (Figure 2). These are joined by a discrete group of mesenchymal cells localized in the somitocoele at the core of the epithelium. This localized EMT endows the embryo with the ability to provide sclerotome-derived fibroblasts and osteoblasts that encircle the NT, synthesize extracellular matrix, and generate the bone and cartilages necessary to construct the vertebral column [84,85].

Intriguingly, the mechanisms controlling EMT of the ventral somite remain unclear. One would postulate that midline signals emanating from the NT and notochord might actively promote the process by inhibiting expression and activity of genes associated with epithelial properties such as Paraxis and N-cadherin. However, axial organ ablation experiments revealed that EMT does not depend on their influence [86]. In addition, Sonic hedgehog (Shh) from the floor plate and notochord, that along with noggin play a role in early formation, survival and maintenance of sclerotomal properties, is not required for cell dissociation [85]. Moreover, it appears that EMT of the ventral somite is not required for initial sclerotome patterning as sclerotomal markers such as Pax1 appear before EMT. Moreover, Pax1 and Pax9 are normally expressed in mice bearing defects in epithelialization and/or segmentation (e.g., Paraxis and Delta-1 mutants) [87,88,89]. Conversely, EMT occurs normally in Pax1/9 or Nkx3.2 mutants [90,91]. An alternative mechanism might be that, if not actively promoted, ventral somitic EMT is initiated merely due to lack of signals that maintain the epithelial somite state. Wnt6, specifically expressed in the epidermal ectoderm regulates early epithelialization of the paraxial mesoderm [81]. At somewhat later stages corresponding to the onset of ventral dissociation, the somite significantly increases in size, and its ventral half might escape the pro-epithelializing activity of ectodermal Wnt6, hence undergoing EMT.

### 3.3. The Dermomyotome and Its Derivatives

The DM is composed of a central sheet adjacent to the ectoderm and four inwardly curved lips that point towards the underlying myotome (Figure 2). The process of DM EMT begins in the central region and progresses medially and laterally (Figure 2). Timewise, it occurs sequentially, in a first stage the central DM dissociates into an intermediate domain composed of compactly aggregated cells which maintain some of the molecular characteristics of the DM epithelium, such as expression of Pax3 and Pax7; in a second stage, full mesenchymalization is attained and these cells generate the subectodermal dermis while losing Pax expression and acquiring instead dermal markers such as *dermo-1* [92]. Notably, the process of DM dissociation is associated with an abrupt change in the orientation of mitotic spindles, from being parallel to the mediolateral axis of the epithelial DM, most mitoses in the intermediate domain shift to become perpendicular with one daughter cell oriented towards the ectoderm and the other facing the myotome [23,33]. This prefigures the relocation of the dissociating progenitors into opposite directions, both into the subectodermal space to form the dermis and into the underlying myotome to contribute cells that ensure its growth [33]. Importantly, only the apical progenitors that colonize the muscle retain N-cadherin, whereas dermal progenitors lose expression of this adhesion molecule [33,93]. Most notably, generation of both dermis and mitotic muscle progenitors is a property inherent to single DM progenitors suggesting these are at least bi-fated [23]. Thus, while this dramatic change in mitotic orientations is associated with cell fate decisions in the developing DM, it is still unclear whether it is causally related to the process of EMT or alternatively, whether the change in pattern of cell divisions and the ensuing EMT are regulated by distinct mechanisms.

Along this line, transcription of ectodermal Wnt6 coincides with the epithelial phase of the somite and early DM, and its subsequent downregulation corresponds to the time of DM dissociation [81]. How do somite and DM cells communicate with the ectoderm from which they are separated by a basement membrane? Recently, it was shown that dynamic filopodia-like protrusions span the subectodermal space overlying the dorsal surface of the somites and contact the ectoderm [94]. These protrusions are actin- and tubulin-positive and require Rac1 for their formation [94]. Since these filopodia exhibit retrograde trafficking of the transmembrane Wnt-receptor Frizzled-7, it is possible that Wnt6 maintains epitheliality of both the somite as well as the early DM sheet. Along this line, the dissociation of the central DM may not occur solely because of downregulation of ectodermal Wnt6, as it was shown to be triggered by FGF8 stemming from the underlying myotome via a Snail1-dependent mechanism [24]. In addition, neurotrophin-3 from the NT was also found to affect DM dissociation and dermis formation [95], and cDermo1 was reported to subsequently mediate epidermal–dermal interactions that lead to feather bud development [96].

EMT of the DM lips is a later process when compared to the central sheet and their behavior depends on the axial level considered. For instance, the ventrolateral edge at brachial and lumbosacral levels of the axis undergoes cell dissociation induced by hepatocyte growth factor/scatter factor stemming from the limb bud mesenchyme and acting through the Met receptor expressed in the ventrolateral DM progenitors. This triggers EMT and cell migration into the limb primordium where appendicular muscles will form [97,98]. At flank levels of the axis, the ventrolateral DM lips generate striated muscle, smooth muscle and endothelium of adjacent blood vessels. However, if interlimb regions are experimentally exposed to hepatocyte growth factor/scatter factor, myoblasts emigrate to colonize ectopically the lateral plate mesoderm, in further confirmation of the role of this factor in cellular EMT [99]. While initially, single lateral DM progenitors “chose” between the two muscle fates, at somewhat later stages, formation of the striated muscle phenotype prevails [15]. *Foxc2* and *Snail1*, specifically expressed in the early lateral lips during the choice period, are both necessary and sufficient for stimulating EMT of lateral DM progenitors as part of the generation of smooth muscle at the expense of striated myotomal cells. Transcription of these genes is stimulated by BMP and Notch signaling which also positively reinforce each other’s activities [14].

An interesting concept emanating from the precedent data is that inhibiting cellular EMT in the DM results in the formation of differentiated myotomal myocytes [14]. This is further strengthened by the observation that only during their epithelial phase, DM progenitors produce striated myocytes [33]. Conversely, upon DM EMT, the dissociating cells generate mitotic myoblasts but not differentiated myocytes [23,33], and cells of the dorsomedial lip of the DM generate migratory dermogenic progenitors upon treatment with Wnt11 [100]. Finally, the morphogen Shh was found to maintain epitheliality of the DM and myocyte differentiation; premature inhibition of Shh signaling caused DM EMT and myotomes were populated by mitotic myoblasts instead of differentiated myocytes [101]. This suggests that formation of a myotome composed of differentiated myocytes does not involve a process of EMT of the corresponding DM precursors. On the contrary, cells translocating from the epithelial DM into the subjacent myotome maintain N-cadherin throughout the entire process comprising the myotome itself. Furthermore, inhibition of N-cadherin-mediated adhesion interferes with myotome colonization whereas overexpression of N-cadherin promotes muscle differentiation [93]. Taken together, in addition to highlighting the significance of EMT in the development of somites and their derivatives, the examples provided here strikingly illustrate the intimate relationship between EMT and fate acquisition during tissue patterning.

## 4. Conclusions and Future Perspectives

EMT is an ancient and relatively well conserved process throughout evolution, apparent already in drosophila and sea urchin where for example Twist, Snail and Cadherin genes take part in gastrulation, and all the way to different and crucial aspects of vertebrate development [102]. In addition, many signaling pathways trigger EMT and these exhibit similarities between embryonic development and pathological states such a carcinoma metastasis and tissue fibrosis. These include different members of the TGFβ superfamily (e.g., BMPs), Wnts, Notch, EGF, HGF, FGF, HIF, *etc.* (e.g., [102,103,104]).

Having the knowledge of conservation of upstream signaling pathways, of downstream genes coding for transcription factors and of effectors leading to changes in apico-basal polarity, cell adhesion, cell motility and extracellular matrix assembly, we are now able to build functional networks that underlie EMT in different biological systems and compare them between normal and diseased states.

Since cell–cell interactions mediate the availability and duration of responsiveness to ligands, great importance should be given to the tissular context used for investigation, as significant changes (and thus different interpretations) may stem from complex *in vivo* analysis when compared for instance to that performed in isolated tissues or explants. In addition, many effector proteins exhibit a heterogeneous subcellular distribution (e.g., cadherins and RhoGTPases), imposing the need for a careful planning of experiments that considers this spatial and temporal dynamics both in single cells and at the level of whole tissue behavior.

More generally, translating insights on EMT and MET processes from development biology to pathological states will advance the field and help realize its full potential in drug discovery, preclinical models of human disease, and ultimately clinical applications.
